# Morphological features of platelet-rich plasma on acellular vascular scaffolds

**DOI:** 10.1097/JS9.0000000000003020

**Published:** 2025-07-14

**Authors:** Shui-Lan Wu, Jian-Yi Xu, Jing Yang, Jian-Wei Chen, Xu-Heng Sun, Hong-Jing Jiang, Rong-Hua Yang

**Affiliations:** a Department of Burn and Plastic Surgery, Guangzhou First People’s Hospital, Guangzhou, Guangdong, China; b School of Medicine, The Second Affiliated Hospital, South China University of Technology, Guangzhou, Guangdong, China; c Department of Internal Medicine-Cardiovascular,Guangdong Provincial People’s Hospital (Guangdong Academy of Medical Sciences), Southern Medical University, Guangzhou, Guangdong, China; d School of Medicine, South China University of Technology, Guangzhou, Guangdong, China

**Keywords:** acellular vascular scaffolds, decellularized extracellular matrix, macrophage, platelet-rich plasma

## Abstract

**Background:**

Materials for small-caliber vascular stents are deficient, with acellular vascular scaffold (AVS) being a promising solution. Platelet-rich plasma (PRP) sourced from the recipient augments the biocompatibility of vascular graft materials. Through integration of PRP onto AVS materials, our goal is to bolster AVS biocompatibility within the body, elevate the M2/M1 macrophage ratio toward graft materials for low degradation rate, and foster robust heparin attachment onto the surface of AVS materials. We aimed to examine the morphological features of PRP on AVS using electron microscopy.

**Methods:**

Hydrophilicity testing of the material gauged alterations in water absorption post-PRP integration. Hematoxylin and eosin staining was used to assess decellularization levels. The collagen and elastin degradation rates in AVS after subcutaneous transplantation in rats were tracked. Immunofluorescence staining was used to determine the relative abundance of M1 and M2 macrophages. Heparin staining, heparin quantification, platelet adhesion, and whole-blood clotting experiments were performed to probe heparin adsorption onto the material.

**Results:**

The presence of a plasma protein network in the outer membrane layer of AVS was evident. PRP enhanced the water absorption rate of AVS, and freeze-drying had no effect on the suture tension of AVS. The ambient storage temperature of this extracellular matrix material was -80°C. Following PRP addition, heparin staining showed a deeper coloration. The heparin release curve was smoother in the PRP group. Additionally, in the experimental group with PRP, a lower number of platelets adhered after heparin addition, and these blood clots had the least weight.

**Conclusion:**

PRP can increase the M2/M1 ratio in AVS subcutaneously transplanted into rats, decelerating the degradation rate of collagen and elastin. Furthermore, PRP can enhance heparin attachment to the AVS, leading to a more gradual heparin release rate.

## Introduction

Autologous vascular grafting has been considered the gold standard for cardiovascular disease treatment. However, the limited availability of donor vessels and the risk of post-transplantation vascular wall damage restrict its application in the repair and regeneration of small-caliber vessels. The success rate of autologous vascular transplantation is >90%. However, because of underlying diseases and supply and demand issues, approximately 40% of the patients lack suitable autologous vessels^[1]^. Consequently, researchers and clinicians have been investigating for improved methods to address the issues associated with small-caliber vascular lesions. Acellular vascular scaffold (AVS) utilizes allogeneic vessels to remove immunogenic nucleic acids through decellularization, thereby possessing excellent tissue regeneration capabilities^[1]^. Biocompatibility is a pivotal factor in evaluating the success of vascular material transplantation. Vascular graft materials must align with host tissues without triggering notable immune rejection or inflammatory responses. The surface properties of a material significantly influence its hemocompatibility for vascular transplantations^[2]^. Up to 75% of synthetic grafts fail within 3 years of transplantation^[3,4]^. Among these, the mid- to long-term outcomes of the widely used polytetrafluoroethylene grafts remain poor^[5]^. Optimizing the morphology and structure of materials can improve their compatibility with the blood and promote vascular regeneration and repair. Thrombosis and vascular narrowing are the common complications of vascular transplantation. Thrombosis may result in occlusion and functional loss of the transplanted vessels, whereas vascular narrowing can affect blood flow and the long-term stability of the transplanted vessels^[6]^. Hence, the prevention and minimization of thrombus formation are paramount for the success of vascular transplantation^[7]^.

**HIGHLIGHTS**

- This article aimed to identify suitable small-diameter vascular graft materials.
- By adding platelet-rich plasma (PRP) is to improve the degradation rate of decellularized vascular materials *in vivo* and to increase the proportion of M2 macrophages, which are associated with tissue repair.
- PRP acts as a connecting medium for heparin. The PRP-treated group exhibited a slower and more sustained release of heparin compared to the phosphate-buffered saline group.

Platelet-rich plasma (PRP) is abundant in various growth factors and plays a pivotal role in vascular regeneration and repair. Similar to vascular endothelial growth factor, transforming growth factor beta, and platelet-derived growth factor-BB, these growth factors are closely related to biological processes^[8]^. By injecting or applying PRP, these growth factors can be released around the graft material, facilitating vascularization and repair of the surrounding tissues, thus improving the biocompatibility of the graft material^[9]^. PRP has anti-inflammatory and anti-infective properties and its use can mitigate inflammation in the surrounding tissues and lower the incidence of immune rejection reactions. Furthermore, growth factors in PRP can stimulate tissue repair and regeneration, expediting the healing of damaged tissues and further enhancing the biocompatibility of the graft material^[10]^. The growth factors and cytokines present in PRP stimulate the synthesis and deposition of extracellular matrix (ECM), enhancing the mechanical stability and tensile strength of the materials. Consequently, PRP use can enhance the material’s mechanical performance, improving its stability and longevity within the body^[11]^. Several clinical studies have confirmed the effectiveness of PRP in enhancing the biocompatibility of vascular graft materials. A notable decrease in post-transplantation complications, expedited healing, and superior vascular regeneration outcomes have been reported in vascular graft materials treated with PRP. These studies provide compelling evidence for the application of PRP in vascular transplantation^[12]^.

Heparin is frequently used in the preparation for vascular animal transplantation to prevent pre- and postoperative thrombus formation, and exerts its anticoagulant effects by binding to antithrombin III. Preoperative prophylactic administration helps lower the risk associated with transplantation surgery and preserves the patency of the original vessels. Heparin application reduces the occurrence of postoperative thrombotic complications, thereby sustaining the patency and functionality of the transplanted vessels. Moreover, heparin can help mitigate postoperative inflammatory responses and facilitate the healing and repair of transplanted vessels^[13]^. After platelet removal, PRP is rich in various plasma proteins. In theory, this network can enable the controlled release of heparin^[8]^.

Natural ECM proteins typically contain cell-binding sites that facilitate cell adhesion and migration. Fibrin can also be used as a raw material to prepare vascular grafts^[14,15]^. Abdominal aortas were harvested from rats and subjected to decellularization to eliminate major cellular immunogens, resulting in AVS formation. Subsequently, AVS materials were subcutaneously transplanted into rats following treatment with phosphate-buffered saline (PBS) and PRP. We evaluated AVS expression in M1 and M2 cells and the degradation rate of the AVS ECM. We treated the transplanted vascular materials by direct immersion in PRP/PRP+ heparin using vacuum freeze-drying to remove internal moisture. We characterized the release kinetics of heparin over time with and without PRP by quantifying the heparin concentration *in vitro*. The surface attachment of heparin was assessed by examining its morphological features using toluidine blue staining. Additionally, platelet adhesion and whole-blood coagulation assays were performed to validate the efficacy of PRP and heparin. The authors hereby confirm that the experimental design, manuscript preparation, data analysis, and all other aspects of this study strictly adhered to the TITAN 2025 guidelines^[16]^.

### Materials and methods

This study was designed and reported in accordance with the Animal Research: Reporting *In Vivo* Experiments guidelines^[17]^. All experiments were conducted in accordance with the ethical regulations of Guangdong Provincial People’s Hospital, Guangdong Academy of Medical Sciences (approval no. GDREC2019284A).

Sprague-Dawley (SD) rats were purchased from a local experimental animal center. Twenty-two rats were used in our experiment, including six rats (three males and three females) aged 8 weeks and weighing 170–190 g, which were used for abdominal aortic collection to prepare the AVS. The remaining 16 rats, all male and 4 weeks old, weighing 100–150 g, were used for subcutaneous transplantation experiments.

### Preparation of platelet-rich plasma

Whole blood was collected from rat hearts in citrate-coated tubes for anticoagulation. Whole blood was subjected to a two-step centrifugation at 200× g for 15 min and 400× g for 10 min to obtain PRP, and platelets were stained using Wright’s staining (Biosharp, China), according to the manufacturer’s instructions. Platelet counts were determined using a hemocytometer. PRP was subjected to repeated freeze-thaw cycles, followed by centrifugation to remove cellular components, and the resulting liquid fraction was used for subsequent experiments^[8,18]^.

### Extraction and quantification of AVS DNA

Eight-week-old rats (both sexes) weighing 170–190 g were anesthetized using isoflurane (2%) and prepared for surgery (*n* = 6). The abdominal fur was removed, the abdomen was incised, and the abdominal aorta was carefully dissected to obtain as much of the aorta as possible while removing small arterial branches. The rat abdominal aorta was decellularized using a combination of sodium dodecyl sulfate (SDS) and 3-[(3-cholamidopropyl)dimethylammonio]-1-propanesulfonate (CHAPS). This method involves incubating the abdominal aorta in a solution containing SDS and CHAPS to disrupt cell membranes and release cellular components, followed by centrifugation to remove cell nuclei and other cellular debris, resulting in AVS production. Subsequently, the DNA content in the AVS was quantified using appropriate methods, such as spectrophotometry or fluorescence assays^[19]^. The blood vessels were first treated with decellularization solution containing 1000 mL water (H_2_O), 1 M sodium chloride (NaCl), 0.05 M sodium hydroxide, 25 mM ethylenediaminetetraacetic acid (EDTA), and 0.5% CHAPS for 28 h, followed by a 24-h PBS wash. Subsequently, they underwent another round of decellularization with a solution containing 1000 mL H_2_O, 1.8 mM SDS, 1 M NaCl, and 25 mM EDTA for 28 h, followed by another 24-h PBS wash. Each step was performed using a shaker at 90 cycles/min. Finally, the AVS obtained was freeze-dried and stored at -80°C for future use. The DNA content was quantified using a DNA extraction kit (Thermo Fisher Scientific, USA), according to the manufacturer’s instructions.

### Hematoxylin and eosin (H&E) staining of abdominal artery of AVS rats subjected to

After washing the AVS and blood vessels with PBS, they were fixed in 4% paraformaldehyde for at least 30 min. Subsequently, the samples were dehydrated using a graded alcohol series. Following dehydration, the specimens were processed as follows: clearing, embedding in paraffin, sectioning, deparaffinization, hydration, staining, dehydration, clearing, and mounting^[20]^. Finally, the samples were mounted onto slides and observed under a microscope.

### Scanning electron microscopy analysis of AVS

After freeze-drying, the AVS samples were divided into the PBS and PRP groups. The samples were fixed with 2 mL of 2.5% glutaraldehyde solution and rinsed with 0.1 mol/L sodium dimethylaminomethyl phosphonate buffer (pH 7.4) and stored at 4°C for 24 h. On the following day, the experimental materials were immersed in 1% citric acid for 45 min. After thorough washing with a buffer solution, the AVS samples underwent alternating cycles of dehydration and ethyl acetate treatment. Scanning electron microscopy (SEM) was performed using an S-3500 N microscope (Japan)^[21]^.

### Water absorption test for AVS materials

Water absorption tests were conducted on AVS materials treated with PBS and PRP^[22]^. The two experimental groups of AVS were harvested, freeze-dried, and weighed (M, dry weight); subsequently, the samples were immersed in double-distilled H2O, and its weight was recorded as m. The formula used to calculate the water absorption rate of the material is as follows: (m - M)/M.

### Impact of different temperatures on the suture tension of AVS

The vascular scaffold materials from the aforementioned experiments were subjected to various storage conditions for different durations, as described: original vascular material group (Ori), was stored at room temperature (RT), 4°C, -20°C, –80°C, and under lyophilization. Each set of materials was stored under these conditions for 1 week and 1 month. Subsequently, the vascular materials were retrieved for suture tension testing using a 6-0 suture thread^[23]^. This procedure used was as follows: the upper end of the blood vessel was fixed to the stent with a clamp, the other end was sutured with a 6-0 suture thread at a distance of approximately 1.0 cm from the port, a weight was hung on the silk thread, and the final weight of the blood vessel fracture was recorded in grams.

### Subcutaneous implantation of AVS

The AVS was sliced into 0.5 cm × 1-cm pieces, lyophilized, and then incubated in either PBS or PRP solution for 1 h before being subcutaneously implanted on the dorsal side of SD rats. The rats used in this study were purchased from a licensed institution. These 4-week-old male rats, weighing 100–150 g, were randomly assigned to different experimental groups, with three animals in each group. The rats were housed in an animal facility under specific pathogen-free conditions^[24]^. Sixteen rats were used: four in the pilot experiment and 12 in the main experiment, all of which survived. Three rats were assigned to each time point. Three rats were housed per cage and provided with standard rat feed pellets. The cages were lined with highly absorbent wood shavings. A standard 12-h light/12-h dark cycle was maintained, and an ambient temperature of 20°C–22°C was ensured. The rats were anesthetized with 2% isoflurane, and their heart rates were continuously monitored during surgery to maintain a proper isoflurane concentration. The fur on the dorsal region was shaved and disinfected, after which AVS tissue blocks were implanted subcutaneously. All the rats were sacrificed in a carbon dioxide chamber to ensure minimal distress and avoid unnecessary fear or suffering.

### MASSON and Elastica van Gieson staining

The AVS was subcutaneously implanted into the dorsal region of the rats for 1–4 weeks. At various time intervals post-implantation, the grafts were extracted from the subcutaneous tissue of the rats and promptly fixed in 4% formalin, with the fixative volume being at least 10 times that of the material, to ensure the preservation of protein content and other tissue components. Following fixation, the tissue samples were gradually dehydrated in alcohol solutions of varying concentrations (30%, 40%, 70%, 90%, and 100%). Subsequently, the dehydrated tissue samples were immersed in a hyaluronic acid solution to facilitate adequate tissue-paraffin contact before being embedded in paraffin. Sections were prepared for MASSON and Elastica van Gieson (EVG) staining.

### Fluorescent staining analysis of immune macrophages in AVS subcutaneous transplantation in rats

The experimental samples were retrieved from subcutaneous transplantation site at 1, 2, and 3 weeks, and fixed overnight at 4°C using 4% paraformaldehyde. Subsequently, the samples were washed 3–5 times using PBS for 15 min, dehydrated in gradient ethanol, and treated with n-butanol for 1 h. The samples were processed in paraffin at 65°C for 3 h, embedded, and sectioned. Following this, primary and secondary antibody incubations were performed, with the concentrations of primary antibodies against cluster of differentiation (CD) 206 and inducible nitric oxide synthase both set at 1:200 (Abcam, USA).

### Toluidine blue staining

Samples from both experimental groups were dried and deparaffinized. The samples were then hydrated, stained, dehydrated, and cleared according to the experimental procedures. Finally, the cells were stained with toluidine blue^[25]^. The images were captured using an optical microscope. If necessary, slides were sealed for future reference.

### Heparin quantification assay

After forming a complex with methylene blue (Thermo Fisher Scientific, USA), a distinct peak emerged at 664 nm, demonstrating a strong linear correlation with the heparin content^[26]^. Hence, ultraviolet spectrophotometry was used to establish a standard curve, enabling the quantification of sodium heparin. AVS samples that were uniform in volume and weight (approximately 2 cm in length) were divided into three groups. Each group was immersed in PBS containing 500 µg/mL heparin for 24 h. Another three groups were soaked in PRP with 500 µg/mL heparin for 3 h. Subsequently, the materials were placed into a 96-well plate, and 100 µL of PBS solution was added. PBS solution was harvested on days 1, 5, 9, 13, 17, 21, 25, and 29, with all samples pre-stored at -80°C. Subsequently, the PBS solutions obtained from each sample were subjected to a methylene blue reaction, and the corresponding heparin concentration was determined based on the absorbance values.

### Whole blood clotting assay and platelet adhesion test

The experimental design involved distinct groups: the PRP with heparin sodium (PRP + Hep), PBS with heparin sodium (PBS + Hep), PRP-only (PRP), and control without supplemental treatment (PBS). Each group was subjected to the following experiments:

Whole blood clotting assay: Fresh rat whole blood was collected stored in anticoagulant tubes within 12. A 10% calcium gluconate solution (prepared at a 1:10 ratio) was mixed with the blood samples, and the resulting mixture was incubated with 500 μL of recalcified whole blood at RT for 30 min. Subsequently, coagulated blood from each sample was rinsed with distilled water, blotted dry with tissue paper, and weighed.

Platelet adhesion test: PRP solution containing platelets (100 μL) was added to the Eppendorf tubes of each experimental group, incubated for 30 min at 37°C, and gently rinsed with PBS. The number of platelets that adhered to each sample was determined using the lactate dehydrogenase (LDH) assay according to the manufacturer’s instructions. The adhered platelets were mixed with the samples, incubated in the reaction mixture, and the absorbance was assessed after the reaction was complete using a spectrophotometer. The resulting changes in absorbance, attributed to LDH activity, were proportional to the platelet concentration, allowing the calculation of platelet concentration based on absorbance^[27]^.

### Statistical analyses

All reported values were averaged (*n* = 3) and expressed as means ± standard deviations. Significant differences were determined using a two-sample *t*-test assuming equal variance. Values with *P* < 0.05 were considered statistically significant.

## Results

### Experimental process

**Figure 1.**
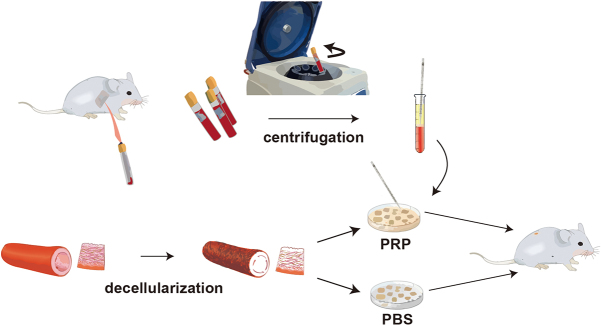
Experiment operation flowchart. Harvest rat heart blood and obtain PRP through centrifugation. Freeze-dry the rat abdominal aorta after decellularization. Subsequently, immerse it separately in PBS and PRP, and then conduct subcutaneous transplantation experiments in rats.

PRP was obtained via a two-step low-speed centrifugation method using rat cardiac blood collected in tubes containing anticoagulant (sodium citrate). Through subsequent experimental verification, the PRP composite quality requirements were obtained. The abdominal aorta of each rat was removed and decellularized. Decellularized AVS was then treated with PBS and PRP. Finally, the rats underwent subcutaneous transplantation for experimental analysis (Fig. 1).

### SEM images, H&E staining, and DNA quantification of AVS

**Figure 2.**
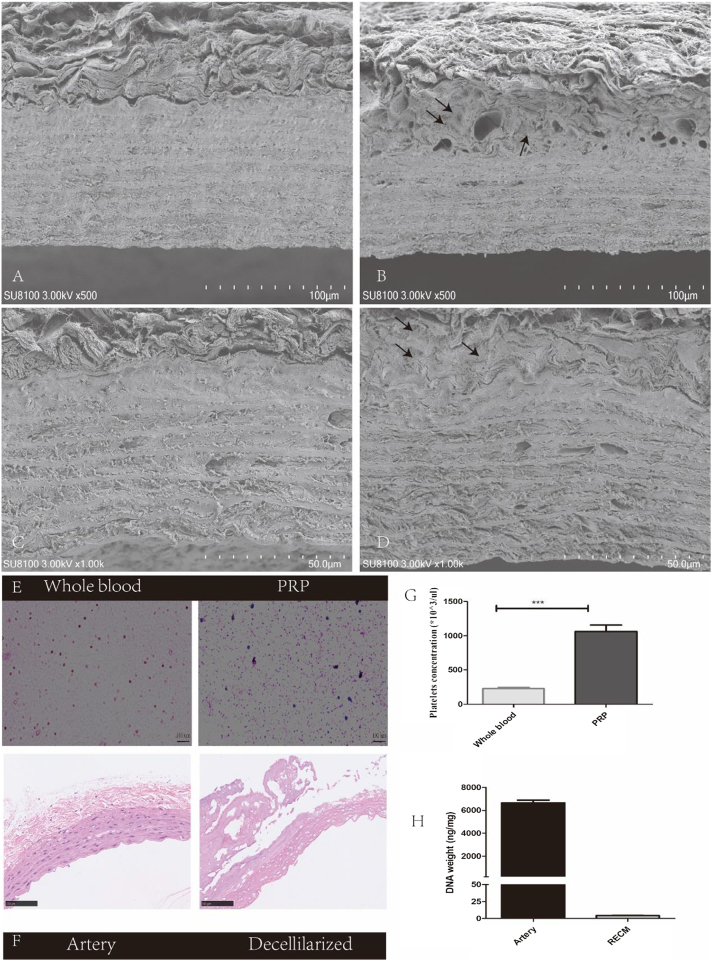
Material characterization. (A, C) Scanning electron microscopy cross-sectional view of decellularized vascular scaffold material treated with PBS. (B, D) Scanning electron microscopy cross-sectional view of decellularized vascular scaffold material treated with PRP. (C) Reticulin staining of PRP and whole blood. (E) Cross-sectional view of intact rat abdominal aorta before decellularization (F-left). Cross-sectional view of rat abdominal aorta after decellularization (F-right). (G) Platelets concentration of PRP and whole blood. (H) DNA quantification results graph. Scale bar 100 µm, arrows indicate fibrous proteins. ***P < 0.001.

The SEM analysis of the decellularized vascular scaffold materials revealed a densely packed structure of regularly arranged layers, with a relatively loose outer membrane (Fig. 2A and C). After PRP treatment, fibrous protein-like structures were visible within the pores of the outer membrane, whereas the protein structures in the middle membrane were indiscernible (Fig. 2B). On further magnification, a protein network-like structure was observed on the outer layer of AVS (Fig. 2D). Reticulin staining revealed that the granular blue-stained area of PRP was larger than that of whole blood (Fig. 2E). Statistical analysis revealed that the platelet count in PRP was 4.66 ± 0.24 times that of platelets in whole blood (Fig. 2G). H&E staining revealed (Fig. 2F, left) that the entire vascular structure displayed distinct layers, including the inner, middle, and outer layers, with cells observed in each layer. As shown in Figure 2F (right), following decellularization, partial disruption of the outer membrane structure was evident, whereas the middle membrane remained relatively intact, with no cellular components observed. The DNA detection result was 3.306 ± 0.589 ng/mg, which is consistent with that of the decellularization standard (Fig. 2H).

### Subcutaneous transplantation of AVS with MASSON and EVG staining

**Figure 3.**
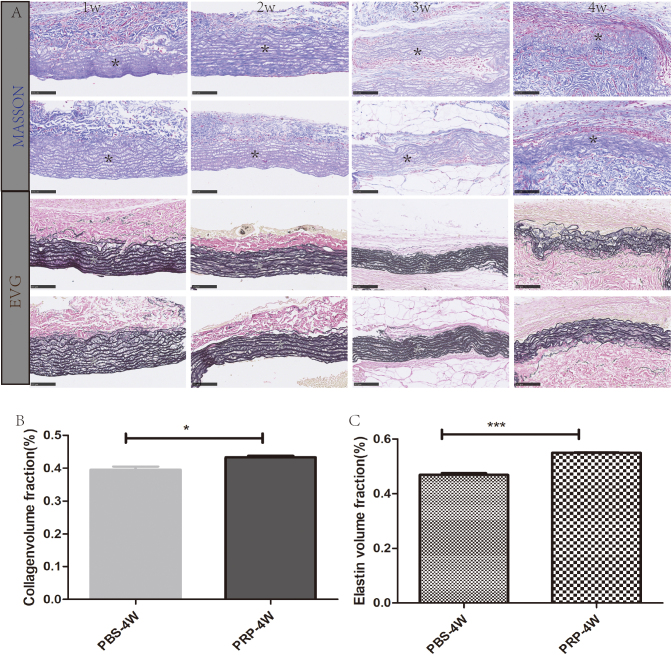
Staining of collagen and elastin in tissues. (A) Results of MASSON staining and EVG staining of AVS subcutaneously transplanted for 1–4 weeks. (B) Quantitative analysis of collagen in the fourth week of MASSON staining. (C) Quantitative analysis of elastic fibers in the fourth week of EVG staining. Scale: 250 µm, *represents the transplantation area, ***P < 0.001.

After subcutaneous transplantation of rat autologous decellularized abdominal aortic vascular scaffold materials for 1–4 weeks, particularly in the fourth week, the structural characteristics of the collagen (blue) and elastic fiber (purple-black) in the PBS group were significantly disrupted, whereas those in the PRP group were relatively intact (Fig. 3A). Quantitative analysis of the blue area in the MASSON staining revealed that the collagen content in the PRP group at the fourth week was higher than that in the PBS control group (Fig. 3B). Quantitative analysis of the purple-black area in EVG staining showed that the elastic fiber content in the PRP group at the fourth week was higher than that in the control group (Fig. 3C).

### Immunofluorescence staining analysis of AVS macrophages

**Figure 4.**
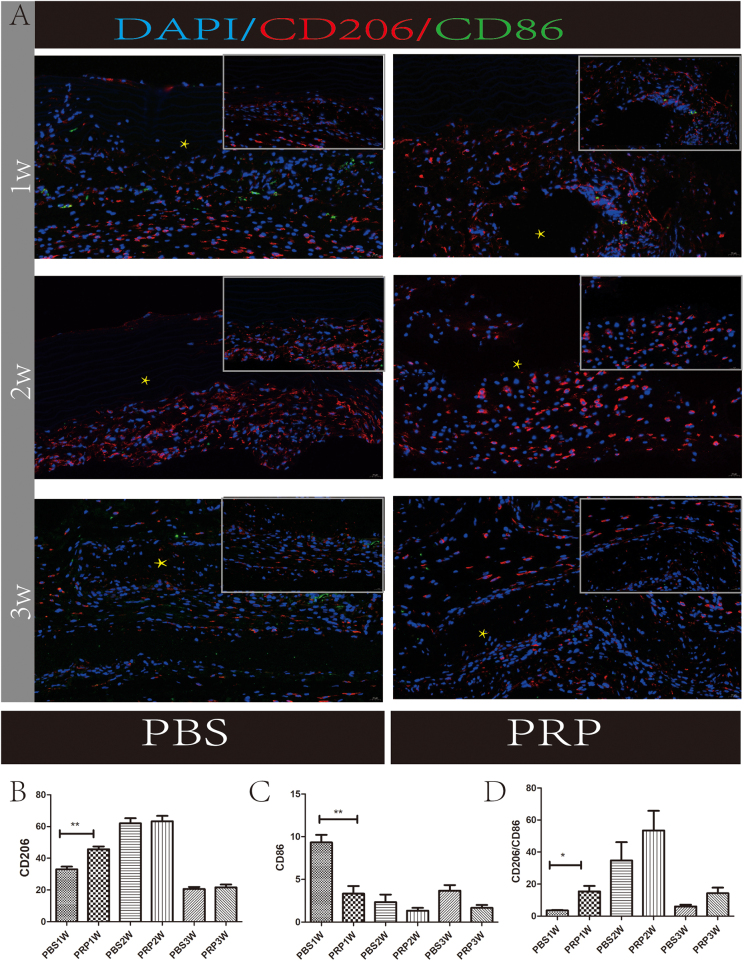
M1 and M2 macrophage tissue staining. (A) Immunofluorescence staining of subcutaneously transplanted AVS from the PBS and PRP groups at different time points. (B) Statistical analysis of CD206 cells in panel (A). (C) Analysis of CD86 cells at different time points in panel (A). (D) Calculation and analysis of CD206/CD86 ratios. Scale: 100 μm. *P < 0.05, **P < 0.01.

To minimize human error, cells in the transplant area were manually counted within a 100 µm-diameter circle across five fields of view, and the average was calculated. The data for CD206 and CD86 cells in these fields of view were statistically analyzed using the ImageJ software. Subcutaneous transplantation of AVS resulted in higher expression of CD86 macrophages and lower expression of CD206 cells in the PBS group than in the PRP group (Fig. 4A–C). Additionally, the M2/M1 ratio was higher in the experimental group than in the control group in the first week (Fig. 4D). By the second week, both experimental groups predominantly exhibited CD206 cells.

### Influence of different temperatures on the suture tension of vascular materials

**Figure 5.**
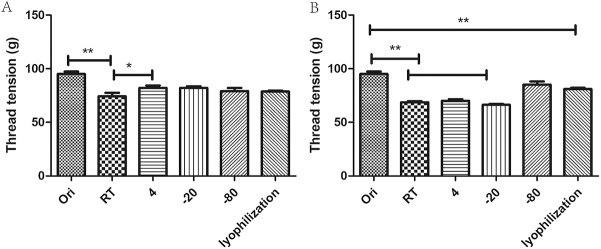
Mechanical testing of AVS. (A) Mechanical testing of vascular materials changes in suture tension of original vascular materials and vascular materials stored under different conditions for 1 week. (B) Changes in suture tension of original vascular materials and vascular materials stored under different conditions for 1 month. *P < 0.05, **P < 0.01.

In the short term (1 week), the impact of temperature on the suture tension of the AVS was minimal, except for a slight decrease observed at RT, which was statistically significant compared with the other experimental groups. The suture tension of the vascular materials decreased slightly compared with that of the original AVS (Fig. 5A). With time, the suture tension of vascular materials stored at RT decreased further. Notable decreases in suture tension were also observed in materials stored at 4°C and -20°C. The suture tension of frozen vascular materials also decreased, with statistically significant differences compared with the original vascular materials, although no statistical difference was observed compared with -80°C. Among the five experimental groups, -80°C demonstrated good preservation of the mechanical properties (Fig. 5B). Therefore, short-term storage temperatures of 4°C, -20°C, and -80°C or freeze-drying followed by storage at -80°C were appropriate. However, for long-term storage (≥1 month), freeze-drying the materials or storing them at -80°C without freeze-drying was preferable.

### Toluidine blue heparin staining, heparin release, whole blood clotting, and platelet adhesion experiments

**Figure 6.**
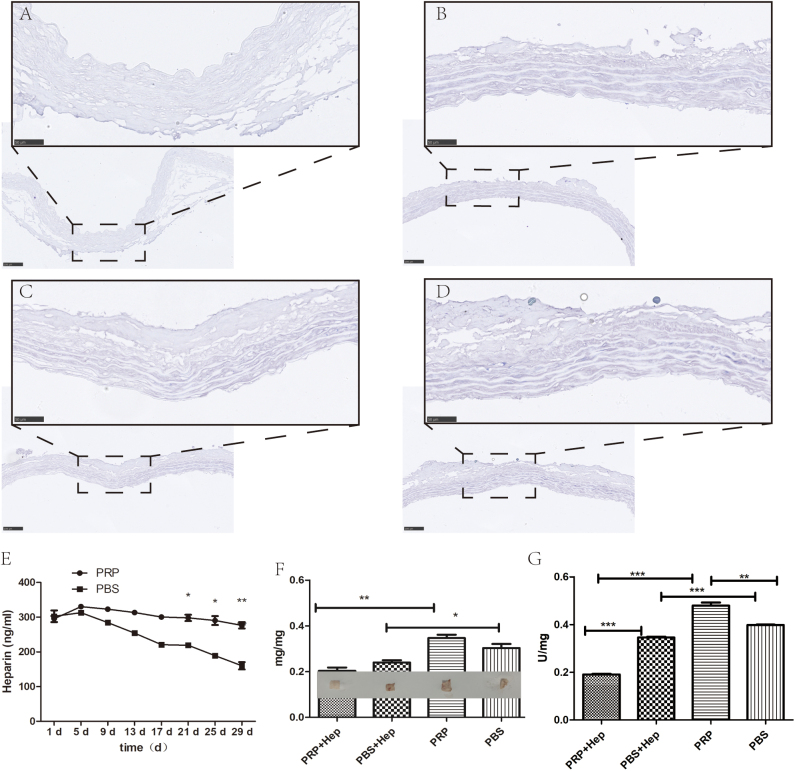
Heparin binding ability test. (A, B) Microscopic images of PBS and PBS-Hep groups after toluidine blue staining. (C, D) Microscopic images of PRP and PBS-Hep groups after toluidine blue staining. (E) Heparin release curves of PBS and PRP groups with heparin added within 1 month. (F) Statistical and overall weight graph of whole blood clotting experiment. (G) Results graph of platelet adhesion experiment. *P < 0.05, **P < 0.01.

Toluidine blue staining showed that the blue-stained area in the PBS group without heparin was relatively mild (Fig. 6A). In the experimental groups treated with PRP or heparin, purple staining was observed, which was difficult to discern with the naked eye (Fig. 6B–D). Heparin release experiments revealed that heparin was detected in both experimental groups within 1 month. Although the PBS group exhibited a decreasing trend, the overall release curve for the PRP group was relatively stable. However, the dose of heparin released also showed a decreasing trend after 2 weeks. From day 17, the amount of heparin released gradually exceeded that in the PBS group (Fig. 6E). The overall and statistical results of the whole blood clotting experiment are presented in Figure 6F. Treating vascular materials solely with PRP increased the weight of blood clots, which was significantly different from that in the PRP-heparin experimental group. The weight of blood clots in the PBS group was lower than that in the sole PRP group, but these differences were insignificant. Both experimental groups with heparin had significantly lower blood clot weights than those without heparin. Similar to the whole blood clotting experiment, the results of the platelet adhesion experiment showed that the PRP-only group had the highest platelet adhesion, which was significantly different from the other experimental groups. However, after addition of sodium heparin, the PRP-Hep group exhibited the lowest platelet adhesion (Fig. 6G).

## Discussion

Currently, the methods for vascular tissue decellularization mainly include mechanical, chemical, and enzymatic decellulari-zation^[3,28,29]^. Dual-detergent decellularization is a chemical decellularization method. The combination of SDS and CHAPS effectively disrupted the cell membrane and facilitated cellular dissolution, thereby achieving complete cell removal. This efficiency contributes to the minimization of residual cells, thereby enhancing the quality and purity of the decellularized tissue. Compared with other chemical agents, they exhibit lower cytotoxicity and milder effects^[30,31].^ Based on our experiments, we found that this decellularization method could effectively remove all cells from blood vessels, while minimizing damage to the vascular endothelium. DNA content analysis revealed that our results were significantly lower than the safety threshold of 50 ng/mg, as described previously^[32]^.

Testing the mechanical properties of arteries and veins in animal models after freezing has no significant impact on vascular mechanical properties^[33]^. At RT, the activities of microorganisms and various enzymes increase, leading to a gradual alteration in the spatial structure of proteins over time^[34]^. In our study, we found that within 1 week of storage, the suture tension of vascular materials placed at 4°C, -20°C, after freezing, or directly stored at -80°C had minimal impact. However, low-temperature storage appeared to be more suitable for vascular material. This observation may be related to the rate of protein degradation in vascular material. Low-temperature conditions are conducive for preserving the integrity of ECM proteins because they can slow down the activity of proteolytic enzymes, thereby protecting proteins from degradation^[35]^.

PRP is widely used in tissue engineering^[36–38]^. Owing to its excellent biocompatibility, PRP can be obtained from autologous sources and harvested in small quantities for multiple uses. PRP mainly contains various plasma proteins. In our previous study, we found that fibrinogen in PRP had a significant impact on cell adhesion. Plasma proteins in PRP form a network-like structure that provides more contact space for cells^[8]^. PRP can be obtained autologously; hence, this simple procedure allows the material to be coated with the patient’s own substances before vascular transplantation. We hypothesized that this approach may effectively reduce the recognition of the material by M1 macrophages, thereby slowing its degradation rate and providing a larger time window for *in vivo* regeneration of the material. Zhu *et al*^[39]^ found that after PRP treatment, the levels of inflammatory cytokines interleukin (IL)-17 and IL-1β were significantly reduced. AVS was performed via subcutaneous transplantation. The results revealed that the PBS-only group exhibited a higher number of M1-CD86 cells and a lower number of M2-CD206 cells during the first week. By comparing the M2/M1 ratios, we found that in the first week, the PRP group was primarily comprised of reparative M2 cells. From the second week onward, both experimental groups predominantly comprised reparative macrophages, with fewer M1 macrophages observed. This further demonstrates the beneficial effect of PRP in promoting M2-M1 conversion during AVS transplantation, thereby reducing the degradation rate of AVS materials. We also hypothesized that the protein network structure of PRP could better adsorb heparin, providing new insights into heparin treatment of vascular graft materials. Through our study, we found that PRP + Hep sustainably released heparin within 30 days, and that the release curve of heparin after PRP treatment was more stable than that after treatment with PBS + Hep alone. Toluidine blue primarily stains cells by combining its cations with acidic substances of tissues^[40]^. Heparin staining experiments revealed that apart from the mild coloration observed in the PBS-only group, all other experimental groups exhibited areas of light blue staining. This observation may be related to a substance present in the PRP that can also bind to toluidine blue. However, whole-blood clotting experiments and platelet adhesion assays revealed that PRP increased platelet adhesion. This effect was counteracted by the addition of heparin, which reduced clotting and platelet adhesion. Through these experiments, we incorporated PRP into vascular scaffold materials, improving their biocompatibility and simplifying the process for heparin treatment, thereby increasing the stability of heparin release on the material surface. Our experiments have some limitations. We did not design specific experiments to explore the mechanism of action of this process; moreover owing to time constraints, we did not validate its effectiveness through in situ transplantation experiments in rats.

## Conclusion

After decellularization, the native vascular scaffold material from the rat removes cellular components from the abdominal aorta while preserving the AVS structure. Our findings from the whole blood clotting experiments and platelet adhesion assays suggest that PRP treatment enhances the ability of vascular material to bind heparin. This study highlights the potential role of PRP in the *in vivo* transplantation of vascular materials and provides valuable data to support the future transplantation of allogeneic or heterologous vascular stent materials. However, our study has certain limitations. We have not been able to improve the data on vascular end-to-end anastomosis in situ transplantation; furthermore, we have not identified the specific mechanism of action of PRP, which will be addressed in our future work.

## Data Availability

Data during the current study will be made available from the corresponding author on reasonable request.

## Originality of content

The authors verify that all information and materials in the manuscript are original.
